# The Role of Circular RNAs in Cardiac Fibrosis: Mechanisms and Therapeutic Implications

**DOI:** 10.1002/clc.70502

**Published:** 2026-11-13

**Authors:** Toufik Abdul‐Rahman, Mrinmoy Kundu, Poulami Roy, Jann Ludwig Mueller‐Gomez, Aditya Gaur, Mohammad Alabdulrahman, Hala Ibrahim Thaalibi, Syeda Asna Shireen Munawer, Emmanuella Ifeoma Okonkwo, Ayaan Hassan Mohamed, Riddhi Mondal, Rakhshanda Khan, Bibek Shrestha, Enaholo Tobechukwu Mathew Olumese, Carl J. Lavie, Rahul Gupta

**Affiliations:** ^1^ Department of Research Toufik's World Medical Association Sumy Ukraine; ^2^ Institute of Medical Sciences and SUM Hospital Bhubaneswar India; ^3^ Department of Medicine North Bengal Medical College and Hospital Siliguri India; ^4^ Center for Research in Health Sciences (CICSA), Faculty of Medicine Anahuac University North Campus Huixquilucan Mexico; ^5^ Yeovil District Hospital Somerset NHS Foundation Trust Yeovil UK; ^6^ Department of Surgery RCSI University of Medicine and Health Sciences Dublin Ireland; ^7^ Beirut Arab University Faculty of Medicine Beirut Lebanon; ^8^ The Tele‐Geriatric Research Fellowship (TGRF) Program East Lansing Michigan USA; ^9^ George Emil Palade University of Medicine, Pharmacy Science and Technology (UMFST) Târgu Mureș Romania; ^10^ University of Nairobi Nairobi Kenya; ^11^ Department of Internal Medicine Jagannath Gupta Institute of Medical Sciences and Hospital Kolkata India; ^12^ Ayaan Institute of Medical Sciences Moinabad India; ^13^ Tribhuvan University Teaching Hospital Kathmandu Nepal; ^14^ Department of Cardiology Ochsner Clinic Foundation New Orleans Louisiana USA; ^15^ Department of Cardiology St. Luke's University Health Network Bethlehem PA

**Keywords:** cardiac fibrosis, circularRNAs, diagnostic biomarkers, therapeutic targets

## Abstract

**Background:**

Cardiac fibrosis, characterized by excessive extracellular matrix (ECM) deposition, contributes to adverse cardiac remodeling and heart failure. Circular RNAs (circRNAs) have emerged as regulators of fibrotic processes, with potential applications in cardiovascular diagnosis and therapy. This review examines the molecular mechanisms through which circRNAs regulate cardiac fibrosis and evaluates their potential as diagnostic biomarkers and therapeutic targets.

**Methods:**

A comprehensive literature search was conducted using PubMed and Scopus to identify studies investigating the roles of circRNAs in cardiac fibrosis, including their molecular mechanisms, diagnostic potential, and therapeutic applications. Relevant experimental and clinical studies were examined.

**Results:**

Experimental evidence implicates circRNAs in cardiac fibroblast activation, myofibroblast differentiation, and ECM remodeling through microRNA‐mediated regulation and interactions with profibrotic signaling pathways, including TGF‐β/SMAD and Wnt/β‐catenin. circSMAD4, circHelz, and circHIPK3 have been associated with profibrotic responses, whereas circNFIB and circCDR1 have demonstrated antifibrotic effects in experimental models. Modulation of circRNA expression has attenuated fibrosis and improved cardiac function in selected animal studies. The structural stability and tissue‐specific expression of circRNAs also support their investigation as potential circulating biomarkers. However, clinical evidence remains limited, and therapeutic translation faces challenges related to targeted delivery, off‐target effects, immunogenicity, and tissue specificity.

**Conclusions:**

CircRNAs are important regulators of cardiac fibrosis and represent promising candidates for diagnostic biomarker development and targeted antifibrotic therapy. Despite encouraging preclinical findings, further clinical research is needed to establish their diagnostic validity, safety, and therapeutic efficacy in humans.

## Highlights

- circRNAs have emerged as promising biomarkers and therapeutic regulators in cardiac fibrosis.
- Gene modulation strategies targeting circRNAs can attenuate fibrotic progression and cardiac remodeling.
- Integrating molecular and computational approaches may accelerate the clinical development of circRNA‐based therapies.

## Graphical abstract

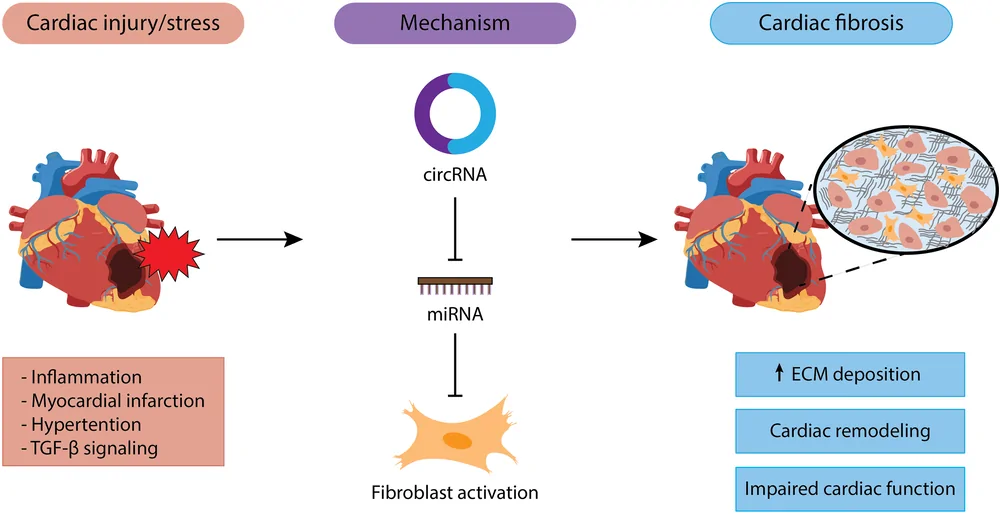

**Abbreviations**
CFs: Cardiac fibroblasts
circRNAs: circular RNAs
ECM: Extracellular matrix
MI: Myocardial infarction
ncRNA: non‐coding RNA
RBPs: RNA‐binding proteins RBPs
TAC: transverse aortic constriction
TGF‐β: Transforming growth factor‐β
α‐SMA: α‐smooth muscle actin

## 1 Introduction

Cardiac fibrosis, defined by the excessive accumulation of extracellular matrix (ECM) in the myocardium, is a hallmark of numerous cardiac pathologies and a major contributor to global health burdens [1]. While fibrosis can be reparative, such as following myocardial infarction (MI), where it replaces dead cardiomyocytes with a collagen‐based scar to maintain structural integrity and prevent catastrophic complications like cardiac rupture, it also significantly contributes to adverse outcomes in various myocardial diseases [2]. These include heart failure with reduced or preserved ejection fraction, genetic cardiomyopathies, and diabetic heart disease. Cardiovascular disease, driven in part by cardiac fibrosis, remains the leading global cause of mortality, accounting for approximately 17.9 million deaths annually, or 32% of all deaths worldwide [3]. The prevalence of cardiac fibrosis is notable, reaching 15% in nonischemic and 30% in ischemic cardiomyopathies [4]. It exacerbates existing conditions, as observed in nonischemic dilated cardiomyopathy, where patients with fibrosis experience a 16.2% higher rate of mortality compared to those without fibrosis [5]. Despite its widespread impact, early detection and management of cardiac fibrosis pose significant challenges due to its asymptomatic nature in early phases and the limited reliability of current diagnostic tools, including cardiac imaging and circulating biomarkers, in identifying subtle fibrotic changes [6].

In recent years, growing evidence has highlighted the importance of non‐coding RNAs (ncRNAs) as key regulators of processes driving cardiac fibrosis [7]. By modulating gene expression at transcriptional and post‐transcriptional levels, ncRNAs orchestrate signaling pathways that drive fibroblast differentiation, ECM deposition, and pathological cardiac remodeling. Dysregulation of several ncRNA classes—including circular RNAs (circRNAs), microRNAs, and long ncRNAs—has already been implicated in fibrotic cardiovascular diseases, suggesting that RNA‐mediated regulatory mechanisms represent an important layer of control in fibrosis development [7]. Among the ncRNAs, circRNAs have gained significant attention due to their unique characteristics [8]. Structurally, circRNAs are distinct due to their covalently closed‐loop configuration, formed by the covalent joining of their 3′ and 5′ ends. This circular structure can comprise only exonic sequences (3′–5′ linkages), intronic lariats (2′–5′ linkages), or exonic sequences with retained introns (3′–5′ linkages) [9]. The closed‐loop structure renders circRNAs highly stable, as it protects them from exonucleolytic decay [10, 11, 12].

Current evidence suggests that circRNAs may act as miRNA sponges, regulate transcription, or interact with RNA‐binding proteins (RBPs) to modulate fibroblast activation and ECM deposition in cardiac fibrosis [13, 14, 15]. However, despite these insights, a comprehensive synthesis of circRNAs’ diverse mechanisms in cardiac fibrosis and their potential as therapeutic targets is still lacking. This review aims to identify key circRNAs implicated in the pathogenesis of cardiac fibrosis and their interactions with known regulators, and to elucidate the molecular mechanisms by which circRNAs influence fibrotic processes. Additionally, it evaluates the therapeutic potential of targeting circRNAs, considering their stability, tissue specificity, and unique molecular interactions. Understanding the role of circRNAs in cardiac fibrosis could enable the development of non‐invasive biomarkers for early detection and disease monitoring, targeted therapies using synthetic inhibitors or mimics to modulate pathogenic circRNA activity, and innovative gene‐editing technologies like CRISPR‐Cas13 for selective circRNA modification. Furthermore, this knowledge could facilitate personalized medicine approaches, tailoring treatments to patient‐specific circRNA expression profiles to optimize therapeutic outcomes.

## 2 General Mechanisms Underlying Cardiac Fibrosis

### 2.1 Cellular Mechanisms

#### 2.1.1 Fibroblast Activation

Cardiac fibroblasts (CFs) are essential for maintaining the ECM and structural integrity of the heart under normal physiological conditions [16]. Following cardiac injury, CFs transition to an activated state characterized by increased proliferation and secretion of ECM components such as collagen and fibronectin [17]. This activation is driven by mechanical stress, inflammatory cytokines, and neurohumoral factors [18], resulting in notable changes, including the expression of α‐smooth muscle actin (α‐SMA) [19]. While the initial response preserves myocardial structure and prevents ventricular dilation, prolonged activation leads to pathological ECM accumulation, increasing myocardial stiffness and contributing to heart failure [20]. Additionally, activated fibroblasts release pro‐fibrotic mediators that perpetuate a feedback loop, recruiting more inflammatory cells and exacerbating fibrosis [21].

#### 2.1.2 Myofibroblast Transdifferentiation

Myofibroblasts arise from the transdifferentiation of fibroblasts in response to injury and stress signals [20]. These cells exhibit a hybrid phenotype, combining properties of fibroblasts and smooth muscle cells, including contractile activity mediated by α‐SMA expression [19]. Myofibroblasts play a dual role: they enhance ECM deposition to reinforce damaged tissue but also contribute to maladaptive remodeling when their activity persists [22]. Collagen types I and III are predominantly produced, leading to increased tissue stiffness and impaired cardiac function [17]. Persistent myofibroblast activity is implicated in arrhythmogenesis, reduced diastolic compliance, and chronic HF [20]. Recent evidence suggests that myofibroblasts may undergo apoptosis or reversion to a quiescent state under certain conditions, highlighting their plasticity and potential as therapeutic targets [23].

#### 2.1.3 ECM Remodeling

The ECM provides structural support to cardiomyocytes and regulates intercellular signaling [24]. Cardiac injury disrupts ECM homeostasis, leading to excessive collagen deposition and alterations in proteoglycans, glycoproteins, and matricellular proteins [25]. Key mediators of ECM remodeling include matrix metalloproteinases (MMPs), which degrade ECM components, and their inhibitors, tissue inhibitors of metalloproteinases (TIMPs) [21]. Dysregulation of the MMP‐TIMP balance results in unchecked ECM accumulation, contributing to fibrosis [26]. Collagen cross‐linking, mediated by enzymes such as lysyl oxidase (LOX) and transglutaminase, exacerbates tissue stiffness, impairing both systolic and diastolic function [18]. This pathological remodeling disrupts cardiomyocyte‐ECM interactions, impairing mechanoelectric coupling and increasing the risk of arrhythmias [19].

### 2.2 Molecular Signaling Pathways

#### 2.2.1 Transforming Growth Factor‐β (TGF‐β) Signaling

TGF‐β is a pivotal regulator of fibrosis, orchestrating fibroblast activation, myofibroblast differentiation, and ECM deposition [27]. Canonical TGF‐β signaling involves the phosphorylation of SMAD2/3, which forms complexes with SMAD4 and translocates to the nucleus to regulate fibrotic gene expression [28]. Key targets include α‐SMA, collagen types I and III, and tenascin‐C [29]. Non‐canonical TGF‐β pathways, including MAPKs (ERK, JNK, and p38), Rho‐GTPases, and PI3K/AKT, further amplify fibrotic responses by enhancing fibroblast proliferation, migration, and collagen synthesis [30]. TGF‐β also inhibits ECM degradation through upregulation of plasminogen activator inhibitor‐1 (PAI‐1) and TIMPs, tipping the balance toward excessive matrix accumulation [17]. Dysregulated TGF‐β signaling is implicated in MI, pressure overload, and diabetic cardiomyopathy, underscoring its therapeutic importance [29].

#### 2.2.2 MAPK Pathways

The MAPK family, comprising ERK, JNK, and p38, plays a critical role in mediating fibrotic responses to stress and injury [23]. These pathways are activated by mechanical stress, cytokines, and growth factors, including TGF‐β [31]. p38 MAPK, in particular, promotes fibroblast activation and collagen deposition [27], while ERK regulates cell survival and migration [30]. JNK signaling is involved in the inflammatory responses associated with fibrosis [28]. Crosstalk between MAPK pathways and TGF‐β signaling exacerbates ECM deposition and fibroblast proliferation [17]. Inhibition of MAPK pathways has demonstrated efficacy in reducing fibrosis and improving cardiac function in preclinical models, highlighting their therapeutic potential [29].

#### 2.2.3 WNT/β‐Catenin Pathway

Cardiac fibrosis is driven by the activation of α–SMA–expressing cells derived from fibroblasts post‐injury and myofibroblasts, which are central to excessive ECM deposition and inflammatory modulation. The WNT signaling pathway is one of the pivotal regulators of these cells in cardiac fibrosis (Figure 1) [32, 33]. The WNT/β‐catenin signaling is activated in fibrotic regions. TGF‐β induces WNT activation via PI3K/Akt‐mediated GSK3β inhibition, leading to β‐catenin stabilization [34, 35]. Canonical WNT signaling cooperates with TGF‐β/Smad pathways to drive myofibroblast proliferation and ECM synthesis [36, 37]. WNT3a enhances fibroblast migration and α‐SMA expression through Smad2, effects reversed by β‐catenin knockdown [38]. GSK3β also modulates TGF‐β1 signaling via Smad3 interaction, with its inhibition promoting myofibroblast transformation [39]. FGF23 further amplifies β‐catenin activity and TGF‐β expression in CFs [40]. Inhibitors like sFRP1/2 and DKK3 modulate WNT signaling and fibrosis. Loss of sFRP1 enhances fibrotic markers [41], while sFRP2 overexpression reduces fibrosis and improves function [42, 43, 44]. DKK3 attenuates fibrosis by enhancing Ang II degradation via ADAM17 and ACE2 regulation, suppressing GSK3β and β‐catenin nuclear translocation; its knockdown reverses these effects [45, 46].

**FIGURE 1:**
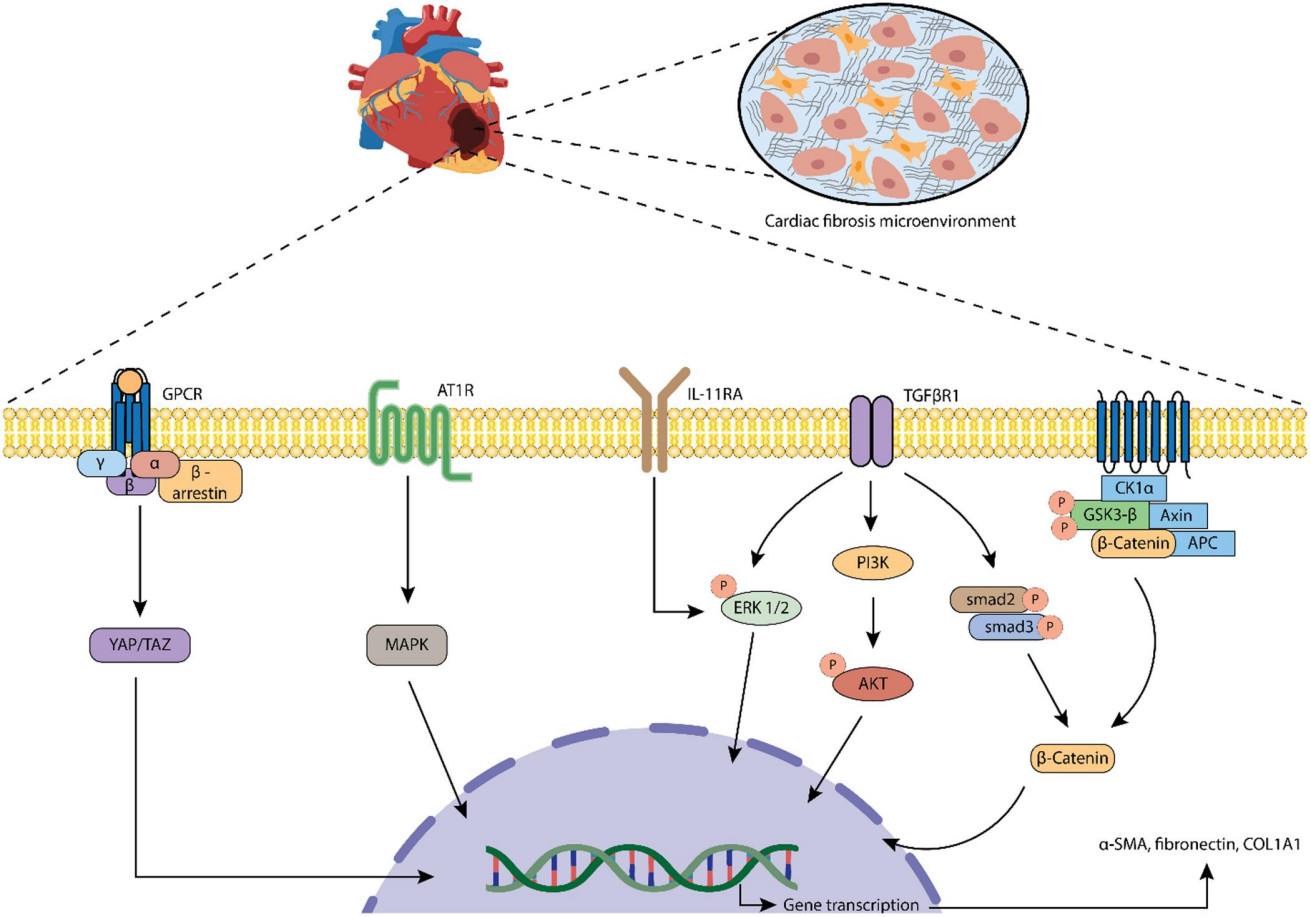
Schematic illustration of molecular signaling pathways in cardiac fibrosis.

### 2.3 Inflammatory Mediators

Chronic inflammation is a driving force in cardiac fibrosis [29]. Following injury, inflammatory cells such as macrophages and neutrophils infiltrate the myocardium and release pro‐inflammatory cytokines, including interleukin (IL)−1β, IL‐6, and tumor necrosis factor‐α (TNF‐α) [47]. These cytokines activate fibroblasts and enhance TGF‐β signaling, creating a pro‐fibrotic environment [48]. Chemokines such as CXCL1 and CXCL8 recruit additional immune cells, perpetuating the inflammatory response [49]. Sustained inflammation leads to excessive ECM deposition, ventricular stiffening, and impaired cardiac function [50]. Targeting inflammatory mediators has emerged as a promising strategy to mitigate fibrosis and improve clinical outcomes in HF [19]. Beyond cytokine‐mediated inflammation, mechanical stress and hypoxia also activate inflammatory signaling pathways that stimulate fibroblasts [20]. Recent studies highlight the role of inflammasomes and Toll‐like receptor pathways in amplifying fibrotic responses, presenting additional therapeutic targets [22]. Among emerging mediators, the heterodimeric complex S100A8/A9, also known as calprotectin, has recently been implicated in cardiac fibrosis. S100A8/A9 functions as a damage‐associated molecular pattern (DAMP) released predominantly by activated neutrophils and monocytes [51]. Extracellular S100A8/A9 interacts with pattern‐recognition receptors such as Toll‐like receptor 4 and the receptor for advanced glycation end products (RAGE), activating downstream nuclear factor‐κB (NF‐κB) signaling and amplifying the production of pro‐inflammatory cytokines [51]. Through these mechanisms, S100A8/A9 contributes to sustained inflammatory responses, leukocyte recruitment, and ECM remodeling, processes that ultimately promote myocardial fibrosis and adverse cardiac remodeling. Emerging evidence also suggests that circulating S100A8/A9 levels correlate with disease severity in cardiovascular conditions such as heart failure, highlighting its potential role as both an inflammatory mediator and biomarker in fibrotic cardiac disease [51].

## 3 Molecular Features and Mechanisms of CircRNAs

### 3.1 Biogenesis and Formation

Linear mRNA is generated from pre‐mRNA exons through alternative splicing, a hallmark process in eukaryotes, whereas circRNAs are formed via backsplicing (Figure 2), a unique mechanism that joins a downstream splice donor site (5′ end of an intron) to an upstream splice acceptor site (3′ end of another intron), resulting in a closed‐loop structure [52]. The process of backsplicing, central to circRNA formation, is facilitated by several splicing mechanisms, including circular lariat‐driven circularization, RNA‐binding protein (RBP)‐mediated circularization, and intronic pairing‐mediated circularization. In circular lariat‐driven circularization, exon skipping forms RNA structures called “lariats,” which are subsequently cleaved internally to generate circRNAs [53]. In RBP‐mediated circularization, specific RBPs such as quaking (QKI), muscleblind (MBL/MBNL1), and fused‐in‐sarcoma (FUS) bind to intronic sequences flanking circularized exons, facilitating their interaction and promoting circRNA formation [54]. Intronic pairing‐mediated circularization relies on complementary base pairing between repetitive sequences in adjacent introns, which aligns the 5′ splice donor site of a downstream exon with the 3′ splice acceptor site of an upstream exon. This alignment allows exon sequences to be retained within the circular RNA through backsplicing, a critical step in circRNA generation [55]. While these mechanisms are well‐recognized, alternative splicing pathways may involve combinations of two or more of these processes. However, further research is required to confirm and elucidate these alternative pathways comprehensively.

**FIGURE 2:**
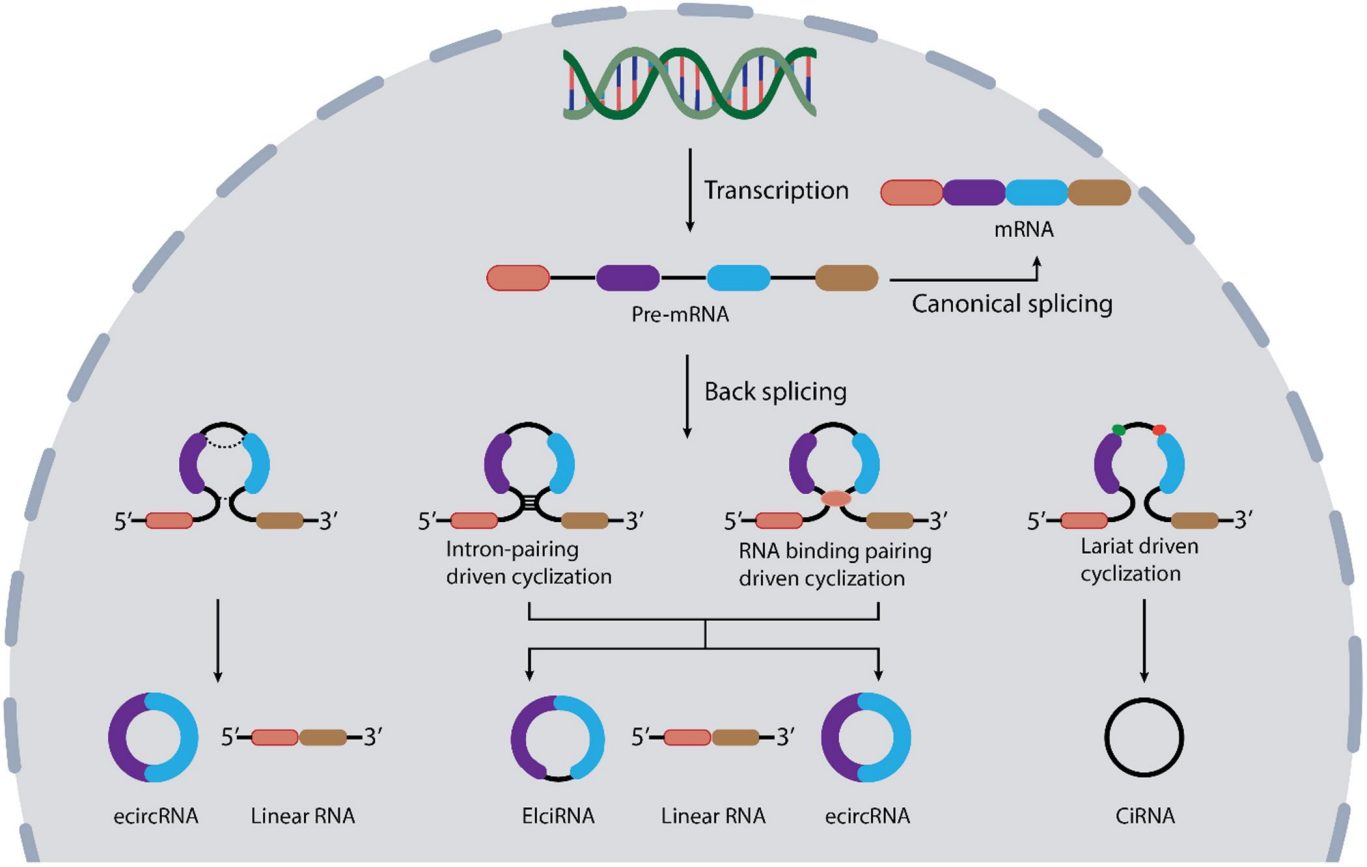
Illustration of circRNA biogenesis.

CircRNAs are classified into four main subtypes: exonic circRNAs (ecircRNAs), the most common type derived primarily from one or more exons; circular intronic RNAs (ciRNAs), composed solely of intronic sequences; exonic‐intronic circRNAs (EIciRNAs), which contain both exonic and intronic regions; and tRNA intronic circRNAs (tricRNAs), formed during pre‐tRNA splicing [10, 52, 56, 57, 58, 59]. EcircRNAs can be produced through lariat‐driven circularization, where a lariat structure forms by the proximity of upstream splice acceptor and downstream donor sites, followed by intron removal and exon joining via a 5′–3′ phosphodiester bond, or through backsplicing facilitated by RBPs that bridge upstream and downstream introns [56, 57, 58]. EIciRNAs are generated through backsplicing similar to ecircRNAs; however, they retain intronic sequences rather than undergoing complete intron removal [60]. ciRNAs arise from intronic lariat structures generated during canonical splicing that escape debranching, thereby forming stable circRNAs and typically accumulate in the nucleus [61]. tricRNAs are created by tRNA splicing enzymes that cleave pre‐tRNA into two parts, forming tricRNAs through a 3′–5′ phosphodiester bond, with the other part producing functional tRNAs [59].

### 3.2 Structural Properties

circRNAs are covalently closed, single‐stranded RNA molecules that lack 5′ caps and 3′ poly(A) tails, forming stable loop structures. This unique configuration confers remarkable resistance to exonuclease‐mediated degradation, distinguishing them from linear RNAs [62, 63]. As a result, circRNAs exhibit extended half‐lives and maintain relatively stable expression levels within cells, even under stressful and inflammatory conditions. Such stability contributes to their persistence in tissue samples and robustness under experimental conditions [64, 65].

A defining structural feature of many circRNAs is the presence of multiple miRNA response elements (MREs), which are complementary to specific miRNAs [66]. In addition, circRNAs frequently contain binding sites for RBPs, enabling interactions with proteins involved in diverse cellular processes [14, 67]. Some circRNAs also harbor internal ribosome entry sites (IRES) or N6‐methyladenosine (m6A) modifications, which facilitate cap‐independent translation by promoting ribosome recruitment [68, 69, 70]. These sequence and structural features underpin the diverse functional capacities of circRNAs.

### 3.3 Functional Properties

A key function of circRNAs is their ability to act as miRNA sponges (Table 1). Through their miRNA response elements (MREs), circRNAs can bind miRNAs with high specificity and affinity, effectively sequestering them from their natural mRNA targets [66]. This interaction prevents miRNA‐mediated repression of target mRNAs, thereby enhancing gene expression. In the context of cardiac fibrosis, such mechanisms may influence pathways such as TGF‐β signaling, contributing to pro‐fibrotic gene regulation [71].

**TABLE 1:** Classification of circular RNAs by functional mechanism.

Functional Category	Mechanism of Action	Representative circRNAs (Cardiac Fibrosis Context)	Biological Impact in Cardiac Fibrosis
miRNA Sponges (ceRNAs) [74].	Bind and sequester microRNAs, preventing them from repressing target mRNAs	circSMAD4	Regulate fibroblast activation, collagen synthesis, and TGF‐β signaling
Protein Scaffolds [75].	Interact with RNA‐binding proteins (RBPs) to modulate their activity or localization	circHelz	Promotes nuclear translocation of YAP and enhances NFATc2‐dependent transcription of pro‐fibrotic genes
Transcriptional Regulators [76, 77].	Interact with transcription machinery or parental gene loci to regulate transcription	circNlgn	Directly regulate transcription of fibrotic genes (ING4, SGK3, collagen) at the chromatin level
Peptide Translation (IRES/m6A‐Driven) [76, 77].	Harbor IRES elements or m6A modifications enabling cap‐independent translation of functional peptides or short proteins	circNlgn	Produce functional peptides in situ that directly modulate fibrotic signaling cascades

Beyond miRNA sponging, circRNAs can interact with RBPs, modulating their activity, localization, or availability. These interactions may affect key cellular processes, including fibroblast activation and ECM deposition. For example, circ‐Foxo3 has been shown to bind proteins such as p21 and CDK2, forming complexes that regulate cell cycle progression, including in CFs [14].

Some circRNAs also exhibit protein‐coding potential. CircRNAs containing IRES or m6A modifications can recruit ribosomes and undergo cap‐independent translation [68, 69, 70]. A notable example is circ‐ZNF609, which is translated into peptides that have been implicated in myoblast proliferation and cellular stress responses [15]. Although the functional relevance of circRNA‐derived peptides in cardiac fibrosis remains under investigation, these findings highlight an additional layer of regulatory potential. CircRNAs localized in the nucleus may contribute to transcriptional and epigenetic regulation. They can interact with transcriptional machinery or chromatin‐modifying complexes, thereby influencing gene expression [72]. It has been proposed that circRNAs may recruit histone acetyltransferases (HATs) or histone deacetylases (HDACs) to specific genomic loci, potentially affecting the expression of fibrosis‐associated genes [73].

In addition, circRNAs can participate in regulatory feedback loops. By sponging miRNAs that target their host genes or components of key signaling pathways, circRNAs may indirectly modulate their own expression or amplify pathway activity. For instance, circRNAs involved in TGF‐β signaling may enhance fibrotic responses through such positive feedback mechanisms [71].

## 4 Dysregulated CircRNAs in Cardiac Fibrosis

Recent research has provided comprehensive insights into the dysregulation of circRNAs in cardiac fibrosis through various experimental approaches. Using a transverse aortic constriction (TAC) mouse model, researchers identified 10 upregulated circRNAs during late‐phase cardiac fibrosis, with three promising candidates, circNAV1, circSMAD4, and circBNC2, showing sequence homologies of 88.7%, 85.0%, and 93.0% with human counterparts, respectively [74]. Among these, circSMAD4 demonstrated unique tissue‐specific expression patterns, with CFs specifically expressing the isoform containing exons 5‐10, while cardiomyocytes expressed other isoforms [74]. This isoform and cell‐type‐specific expression pattern suggest a tightly regulated role in fibrotic remodeling.

In a study investigating circRNA expression in hypertrophy‐associated heart diseases, circITGa9 (hg38_circ_0030600), a novel circRNA derived from the ITGA9 gene, was found to be significantly upregulated by approximately 38‐fold in cardiac remodeling compared to normal myocardium, suggesting a pivotal role in cardiac pathology [78, 79, 80, 81]. Functional analyses (GO and KEGG) revealed that circITGa9 is involved in pathways associated with hypertrophic and dilated cardiomyopathy, as well as cell adhesion, all of which are critical to cardiac remodeling processes. Further research identified 283 differentially expressed circRNAs (DECs) in a TGF‐β1‐induced cardiac fibrosis model, including 79 up‐regulated and 204 down‐regulated variants [82]. These findings indicate that circRNAs are not only differentially expressed but are also closely associated with cell‐type specificity, disease stage, and pathological context, providing important clues for subsequent mechanistic investigation.

## 5 Molecular Mechanisms of CircRNAs in Cardiac Fibrosis

### 5.1 CircRNA‐miRNA Interactions

CircRNAs exert significant influence in cardiac fibrosis by acting as miRNA sponges, as demonstrated by the circSMAD4/miR‐671‐5p/FGFR2 axis. Mechanistically, circSMAD4 acts as a sponge, directly binding to and suppressing miR‐671‐5p (Figure 3), a microRNA with established anti‐fibrotic properties. This suppression leads to the upregulation of FGFR2, a downstream target of miR‐671‐5p, which subsequently drives the differentiation of CFs into myofibroblasts—a key event in fibrotic remodeling [19, 74]. In both in vitro and in vivo models, circSMAD4 significantly promoted fibroblast activation and fibrogenic gene expression. Silencing of circSMAD4 using siRNA (si‐circSMAD4) successfully attenuated fibrosis in a short‐term injury model induced by isoproterenol (ISP) and even reversed established fibrosis in mice subjected to 8 weeks of TAC, highlighting its potential in both early and advanced disease stages. Interestingly, although si‐circSMAD4 did not substantially reverse cardiac hypertrophy, likely due to its selective expression in fibroblasts rather than cardiomyocytes, it did improve cardiac function and reduce chamber dilation and fibrotic burden in late‐stage TAC mice [74].

**FIGURE 3:**
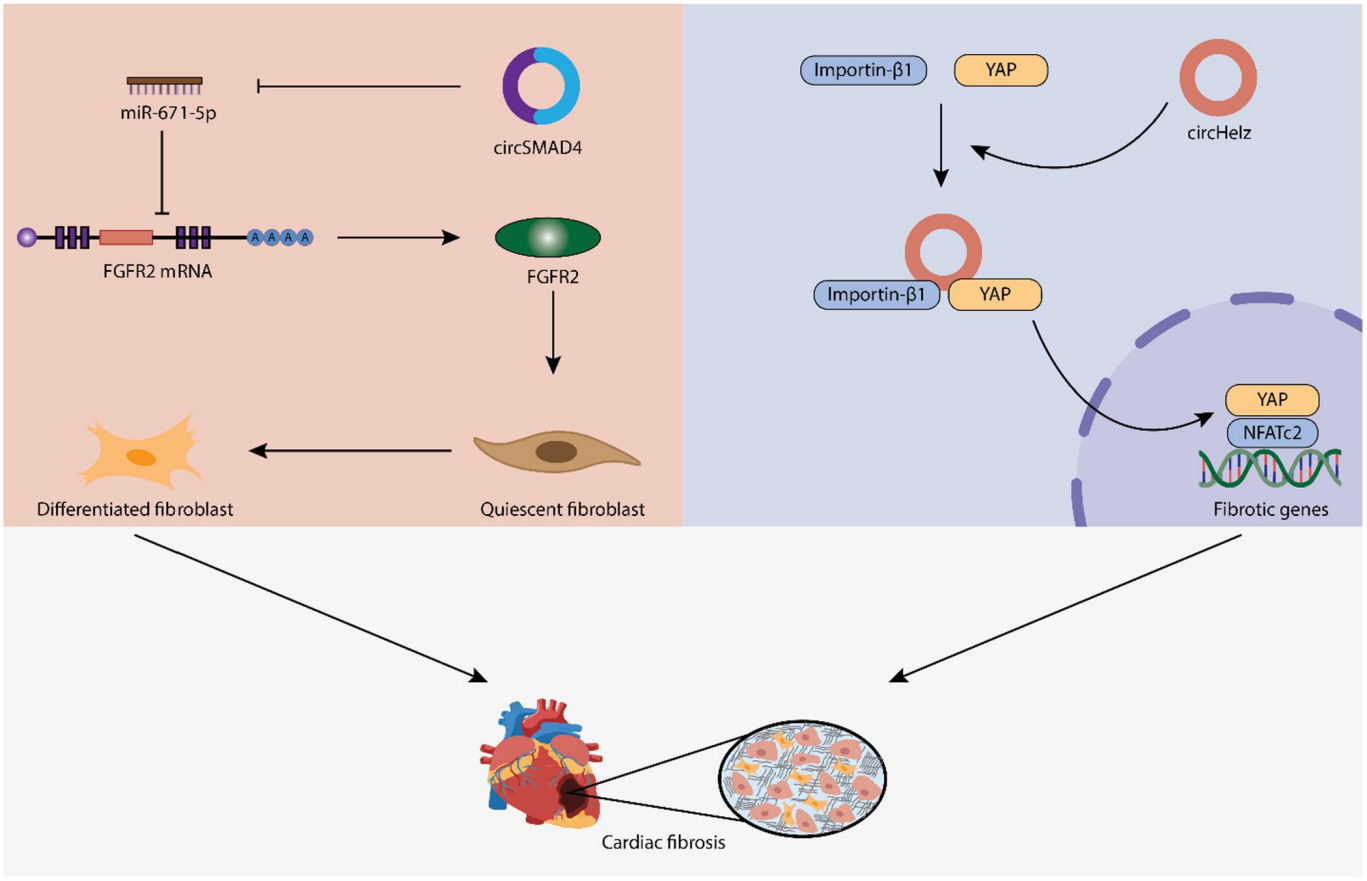
Molecular mechanism of circSMAD4 and circHelz in cardiac fibrosis.

Numerous circRNA–miRNA axes have been identified across diverse pathological cardiac contexts. For instance, circCDR1as exerted anti‐fibrotic effects in pig models by sponging miR‐671‐5p, particularly when treated with natural compounds such as bufalin and lycorine [83]. In myocardial ischemia‐reperfusion injury, circARAP1 aggravated fibrosis by modulating the miR‐379‐5p/KLF9 pathway and promoting Wnt/β‐catenin signaling [84]. Likewise, circPAN3 contributed to autophagy‐mediated fibrosis via the miR‐221/FoxO3/ATG7 pathway [85], and circUbe3a, derived from M2 macrophages, enhanced CF activation through the miR‐138‐5p–RhoC axis [86]. Interestingly, several circRNAs have also been found to inhibit fibrosis. For example, circMACF1 alleviated TGF‐β1‐induced CF activation via the miR‐16‐5p/SMAD7 axis [87], circNFIB reduced CF proliferation through miR‐433 [88], and Circ_LAS1L suppressed fibrotic activity by modulating the miR‐125b–SFRP5 pathway [89].

#### 5.1.1 Cell and Context‐Specific Regulation of miRNA Sponging

CircRNA–miRNA interactions in cardiac fibrosis do not form a simple directional network in which each circRNA uniformly promotes or suppresses fibrosis. Rather, they constitute a complex, context‐dependent regulatory system, in which the functional outcome of any given circRNA–miRNA axis is shaped by multiple factors. These include the specific cell type, as downstream mRNA targets differ substantially between different cell types such as fibroblasts, cardiomyocytes, macrophages, and endothelial cells; the disease model or injury stimulus, since ischemia, pressure overload, diabetic cardiomyopathy, or angiotensin II exposure each create distinct transcriptomic environments that alter competing endogenous RNA (ceRNA) stoichiometry; the temporal stage of fibrosis, with the balance between pro‐ and anti‐fibrotic circRNAs shifting dynamically over the course of injury and remodeling; and species or experimental system differences, as findings from murine TAC models, porcine ischemia models, or in vitro TGF‐β stimulation are not always directly comparable.

A striking example of this principle is provided by circSMAD4 and circCDR1as, both of which sponge miR‐671‐5p yet produce opposite effects on fibrosis. circSMAD4 is predominantly expressed in CFs (specifically the exon 5–10 isoform, circSMAD4‐III), where suppression of miR‐671‐5p leads to upregulation of FGFR2 and activation of pro‐fibrotic myofibroblast differentiation programs [74]. In contrast, circCDR1as exerts anti‐fibrotic effects across broader cardiac cell populations, likely by derepressing the anti‐fibrotic target SEPP1 [83, 90], illustrating that identical miRNA sponging can yield diametrically opposite outcomes depending on downstream target networks and cell type. Other contextual factors further shape these effects. For example, circCDR1as's anti‐fibrotic activity was observed in a porcine post‐infarction model treated with natural compounds (bufalin and lycorine) [83], suggesting that external stimuli can alter miRNA target availability or ceRNA stoichiometry. Moreover, the relative abundance of the circRNA, the miRNA, and competing mRNA targets strongly influences the functional impact, meaning that the same circRNA–miRNA interaction may drive pro‐ or anti‐fibrotic outcomes under different stoichiometric conditions.

This example underscores a fundamental principle in circRNA biology: functional consequences of miRNA sponging cannot be inferred from the interaction alone, but depend on cellular context, downstream regulatory networks, and temporal dynamics. Resolving such apparent contradictions will require comparative studies in the same cell type and disease model, as well as systematic mapping of miRNA targetomes, to fully understand how shared miRNA interactions translate into divergent phenotypes.

### 5.2 circRNA‐Protein Interactions

In addition to miRNA sponging, circRNAs also act as molecular scaffolds, modulating cardiac fibrosis by directly interacting with proteins and influencing their function, stability, or localization. For instance, circHelz was found to be upregulated in MI models and TGF‐β‐treated CFs, where it bound to YAP, promoting its nuclear translocation and activation of NFATc2, thereby exacerbating fibroblast proliferation [75]. Similarly, circITGa9 contributed to myocardial fibrosis by binding to tropomyosin 3 (TPM3) and promoting actin polymerization, linking cytoskeletal reorganization to fibrotic progression [78]. In contrast, circYAP was downregulated in pressure overload models; its interaction with TPM4 and ACTG attenuated actin polymerization and fibrosis [91]. Other protein‐interacting circRNAs also modulate fibrosis through unique mechanisms: DICAR bound VCP to prevent MED12 degradation in diabetic cardiomyopathy [92]; circ‐AMOTL1 enhanced EIF4A3‐mediated MARCKS stability under high‐glucose stress [93]; and circWhsc1 facilitated TRIM59–STAT3 phosphorylation to promote cardiomyocyte proliferation during hypoxia [94].

#### 5.2.1 Context‐Dependent Effects

CircRNA–protein interactions can produce divergent or even antagonistic effects on cardiac fibrosis, depending on the regulatory node, subcellular mechanism, and disease context. A notable example is provided by circHelz and circYAP, which both modulate the YAP signaling axis but at distinct mechanistic levels. circHelz acts as a protein scaffold that promotes nuclear translocation of YAP, enhancing NFATc2‐dependent transcription of pro‐fibrotic genes in the setting of MI and TGF‐β stimulation [75]. In contrast, circYAP functions upstream by interacting with cytoskeletal components (TPM4 and ACTG), suppressing actin polymerization—a prerequisite for YAP mechanosensing and nuclear entry—thereby attenuating fibrosis in pressure‐overload models [91, 95].

Mechanistically, these circRNAs operate at distinct nodes within the same pathway rather than directly competing: circHelz acts post‐translationally on YAP localization, while circYAP acts pre‐translationally via the cytoskeleton. Their relative balance is also disease‐context dependent: circYAP is downregulated in pressure overload, whereas circHelz is upregulated in MI models [96]. This context dependence aligns with evidence that YAP itself exerts cell‐type‐ and context‐specific effects, with fibroblast‐specific deletion attenuating fibrosis post‐MI, and cardiomyocyte‐specific deletion exacerbating fibrosis and mortality [96].

This example illustrates a broader principle: circRNA–protein crosstalk is dynamic and context‐dependent, and multiple circRNAs responsive to the same upstream stimulus such as TGF‐β, can have opposing effects. It is plausible that some circRNAs, such as circYAP, may function as early negative feedback regulators, transiently restraining fibrotic signaling before being overwhelmed during sustained pro‐fibrotic stimulation. These dynamics have important therapeutic implications: interventions aimed at restoring anti‐fibrotic circRNAs may be most effective in early‐stage fibrosis, while advanced disease may require combination approaches to overcome entrenched pro‐fibrotic signaling. Critically, no study to date has performed longitudinal profiling of pro‐ and anti‐fibrotic circRNAs within the same experimental model across the full time course of fibrotic progression [75]. Such studies are essential for determining whether the circRNA landscape shifts in a predictable, stage‐dependent manner that could guide the timing of circRNA‐based therapeutic intervention.

### 5.3 Signaling Pathways Modulated by circRNAs

CircRNAs exert broad regulatory effects on cardiac fibrosis by modulating key signaling pathways, including TGF‐β/Smad, Wnt/β‐catenin, and Hippo/YAP signaling. Rather than acting as primary signaling molecules, circRNAs function as upstream modulators that fine‐tune pathway activity through miRNA and protein interactions (Table 2). Importantly, these pathways do not operate in isolation. Crosstalk between TGF‐β, YAP, and Wnt signaling creates an integrated network that governs fibroblast activation and tissue remodeling. CircRNAs, by targeting multiple components within this network, can amplify or dampen fibrotic responses depending on cellular context.

**TABLE 2:** Network‐level convergence of circRNAs on core pathological pathways.

Pathological Hub	circRNA	Regulation	Specific Axis/Mechanism	Pathological Context	Reference
TGF‐β/Smad Signaling	circSMAD4	Up	circSMAD4/miR‐671‐5p/FGFR2	Promotes fibroblast‐to‐myofibroblast transition	[74].
	circMACF1	Down	circMACF1/miR‐16‐5p/SMAD7	Alleviates TGF‐β1‐induced CF activation	[87].
	circHIPK3	Up	Sponges miR‐29b‐3p	Increases TGF‐β function and α‐SMA expression	[97, 98, 99, 100].
Wnt/β‐catenin Activation	circARAP1	Up	miR‐379‐5p/KLF9 axis	Aggravates fibrosis by promoting Wnt/β‐catenin signaling	[84].
	circCELF1	Up	miR‐636/DKK2 axis	Modulates Wnt signaling by targeting DKK2	[101, 102].
	circ_012164	Up	miR‐9‐5p/DKK2 axis	Influences Wnt‐related fibrotic pathways	[101, 102].
	circIGF1R	Up	DDX5/β‐catenin axis	Activates β‐catenin to enhance cardiac repair	[103].
Cytoskeletal Remodeling	circITGa9	Up	Binds TPM3 protein	Enhances actin polymerization and collagen expression	[78, 104, 105].
	circYAP	Down	Binds TPM4 and ACTG	Attenuates actin polymerization and fibrosis	[91].
Inflammatory/Macrophage Crosstalk	circUbe3a	Up	miR‐138‐5p/RhoC axis	M2 macrophage‐derived; enhances CF activation	[86].
	circHelz	Up	Binds YAP/NFATc2	Promotes YAP nuclear translocation and CF proliferation	[75].
	circNlgn	Up	Encodes Nlgn173 protein	Activates inflammatory cascades and DNA damage	[76, 77].

### 5.4 The Inflammation–Fibrosis Axis: circRNAs as Mediators of Cellular Crosstalk

Cardiac fibrosis is increasingly recognized as resulting from a complex interplay between inflammatory and fibrotic processes. Following myocardial injury, immune cells infiltrate the myocardium and release cytokines and extracellular vesicles that influence cardiac fibroblast behavior, promoting their differentiation into ECM‐producing myofibroblasts [106]. Recent evidence indicates that circRNAs participate in this process not only as intracellular regulators but also as mediators of intercellular communication. Their covalently closed structure confers high stability, and many circRNAs are enriched in extracellular vesicles, including exosomes, enabling them to function as signaling molecules transferred between immune cells and fibroblasts.

A notable example of such immune–stromal communication is circUbe3a, which is present in extracellular vesicles released by alternatively activated M2 macrophages following MI [86]. These vesicles can be internalized by CFs, delivering circUbe3a into recipient cells. Mechanistically, circUbe3a acts as a competing endogenous RNA that sponges miR‐138‐5p, thereby relieving suppression of its downstream target RhoC, a small GTPase involved in cytoskeletal regulation and cell motility. Activation of the circUbe3a/miR‐138‐5p/RhoC axis promotes fibroblast proliferation, migration, and differentiation into ECM‐producing myofibroblasts [107]. In vivo studies further show that increased delivery of circUbe3a‐containing vesicles exacerbates myocardial fibrosis, highlighting the contribution of macrophage‐derived circRNAs to fibrotic remodeling. Beyond mediating direct immune–fibroblast communication, circRNAs can amplify intracellular signaling pathways that sustain fibroblast activation. For instance, circHelz expression is induced by the transcription factor NFATc2 under pro‐fibrotic conditions. Once expressed, circHelz interacts with Yes‐associated protein (YAP1), promoting its nuclear localization and activating transcriptional programs that drive fibroblast proliferation and fibrotic gene expression [75]. Consistent with this mechanism, knockdown of circHelz attenuates fibroblast activation and reduces cardiac fibrosis in experimental models, underscoring its role in maintaining a pro‐fibrotic signaling environment [75].

Collectively, these findings support the concept that circRNAs act as key molecular intermediaries linking inflammatory signaling with fibroblast activation, coordinating both intercellular communication and intracellular amplification of pro‐fibrotic pathways. By enabling crosstalk between immune cells and structural cells of the heart, circRNAs facilitate the transition from acute inflammation to chronic fibrotic remodeling. This perspective has important therapeutic implications: instead of targeting fibroblasts alone, strategies that modulate the immune–fibrotic niche—including circRNA biogenesis, vesicular packaging, or cellular uptake—may provide a more selective means of disrupting maladaptive fibrosis while preserving beneficial post‐injury repair.

## 6 circRNAs as Diagnostic Biomarkers in Cardiac Fibrosis

Several circRNAs have been identified as potential diagnostic biomarkers across a range of cardiovascular conditions, highlighting their broader clinical relevance. In acute MI, circRNAs such as circRNA_104761, circSlc8a1, and circNFIX have been reported as candidate biomarkers, while circRNA_0001946, circZNF609, and circRNA_0005540 have shown diagnostic potential in coronary artery disease [108]. In the context of metabolic cardiac disorders, transcriptomic analyses in diabetic cardiomyopathy have identified approximately 212 differentially expressed circRNAs, with circPHF20L1 demonstrating the most significant upregulation (4.03‐fold change), suggesting its potential utility as a diagnostic indicator [109]. Similarly, studies in diabetes‐related cardiac conditions have shown that DICAR levels in peripheral blood mononuclear cells and plasma are significantly reduced in diabetic patients compared to healthy controls [92], supporting its role as a circulating biomarker candidate. Beyond these conditions, circRNAs have also demonstrated potential for early detection and disease monitoring. Notably, circRNA_0062960 has been proposed as a biomarker for compensated heart failure, circMARK3 for aortic dissection, and circRNA_025016 for atrial fibrillation [108].

These findings underscore the versatility of circRNAs as biomarkers across diverse cardiovascular diseases. However, most of these candidates remain at the discovery stage, with limited validation in large, independent clinical cohorts, emphasizing the need for further studies to establish their diagnostic accuracy and clinical utility.

### 6.1 Human Evidence Supporting circRNAs as Biomarkers

Evidence from human studies provides important support for the clinical relevance of circRNAs in cardiovascular disease. Transcriptomic analyses of myocardial tissue from patients with ischemic heart failure have identified dysregulated circRNA expression profiles, along with circRNA‐centered regulatory networks involving circRNA–microRNA–mRNA interactions. These findings indicate that circRNAs are actively involved in pathways underlying cardiac remodeling and heart failure progression, reinforcing their potential as biomarker candidates. For example, recent studies have characterized circRNA‐centered ceRNA networks in human failing hearts, including those involving circBPTF and multiple regulatory microRNAs [110]. Such networks demonstrate that circRNAs are integrated into disease‐relevant regulatory circuits in human cardiac tissue, rather than representing incidental transcriptional byproducts.

Importantly, these findings confirm that circRNAs are detectable and functionally relevant in human cardiovascular tissues, a prerequisite for their consideration as biomarkers. However, most currently available human evidence is derived from transcriptomic profiling studies or relatively small patient cohorts. Large‐scale, well‐characterized clinical studies—particularly those evaluating circulating circRNAs in accessible biofluids such as plasma or serum—remain limited. Consequently, although circRNA dysregulation in human cardiac disease is increasingly well documented, further investigation is required to determine whether specific circRNAs can serve as robust, reproducible, and clinically actionable biomarkers, particularly for cardiac fibrosis.

### 6.2 Translational Challenges and Validation Gaps

Despite the growing number of circRNA candidates identified in cardiovascular research, clinical validation remains limited. Most circRNAs proposed as biomarkers have been identified through preclinical models or exploratory transcriptomic analyses, rather than large, multicenter clinical studies. Furthermore, many investigations rely on myocardial tissue samples obtained during cardiac surgery or transplantation, which limits the feasibility of translating these findings into non‐invasive biomarkers suitable for routine clinical use.

Several key challenges must be addressed before circRNAs can be implemented as reliable diagnostic tools. First, large‐scale prospective studies are required to validate candidate circRNAs and establish their diagnostic accuracy, sensitivity, and reproducibility across diverse patient populations. Second, the disease specificity of circRNA biomarkers remains uncertain, as many circRNAs participate in regulatory networks that extend beyond cardiac fibrosis to include inflammatory, metabolic, and broader remodeling processes. Third, methodological standardization remains a major limitation: protocols for circRNA detection, normalization, and quantification in clinical samples are still evolving, complicating cross‐study comparisons and limiting reproducibility [108].

These limitations indicate that circRNA biomarker research remains at an early translational stage. Although circRNAs show considerable promise as diagnostic and prognostic tools in cardiovascular disease, robust clinical validation and standardized analytical frameworks will be essential before circRNA‐based biomarkers can be integrated into routine cardiovascular diagnostics.

### 6.3 circRNAs Versus Circulating microRNAs as Diagnostic Biomarkers

The potential of circRNAs as diagnostic biomarkers should be considered in the context of the more established field of circulating microRNA biomarkers, which have been extensively studied in cardiovascular disease. Circulating microRNAs are readily detectable in biofluids such as plasma and serum and have demonstrated the ability to reflect underlying pathological processes, making them attractive non‐invasive biomarkers.

Several studies have evaluated circulating microRNAs as diagnostic tools in heart failure, including heart failure with preserved ejection fraction (HFpEF). Systematic reviews and meta‐analyses have identified specific microRNA panels capable of distinguishing heart failure subtypes with moderate diagnostic accuracy, with reported area under the curve (AUC) values of approximately 0.86 for heart failure with reduced ejection fraction (HFrEF) and 0.79 for HFpEF. These findings suggest that circulating microRNAs can provide incremental diagnostic value alongside conventional biomarkers such as natriuretic peptides [111]. In addition, human clinical studies have shown that circulating microRNA profiles differ between patients with heart failure and healthy controls, and that specific microRNAs—including miR‐1233, miR‐183‐3p, miR‐193b‐3p, miR‐190a, and miR‐671‐5p—can distinguish between heart failure phenotypes [112]. Importantly, several of these microRNAs are also mechanistically linked to cardiac fibrosis and remodeling. For example, miR‐21 is consistently upregulated in failing myocardium and promotes fibroblast activation through ERK–MAP kinase signaling, thereby contributing to interstitial fibrosis [113].

Compared with microRNAs, circRNAs possess several theoretical and emerging practical advantages as biomarkers. Their covalently closed structure confers greater resistance to exonuclease‐mediated degradation, resulting in enhanced stability in circulation. In addition, circRNAs often exhibit higher tissue and cell‐type specificity, which may improve their ability to distinguish between closely related pathological states, such as different forms of heart failure or stages of cardiac fibrosis. However, despite these advantages, circRNAs remain less well validated in clinical settings. While microRNA biomarkers have been evaluated in large patient cohorts and meta‐analyses, most circRNA studies are still limited to exploratory transcriptomic analyses or small‐scale clinical investigations. Moreover, standardized methods for circRNA detection and quantification in biofluids are still evolving, whereas microRNA profiling techniques are comparatively well established.

Overall, these differences highlight a key distinction: microRNAs currently represent clinically more mature biomarkers, whereas circRNAs offer potentially superior stability and specificity but remain in earlier stages of translational development. Future studies directly comparing circRNA and microRNA diagnostic performance—particularly in the same patient cohorts—will be essential to determine whether circRNAs can complement or outperform existing RNA‐based biomarkers. Integrative approaches combining both circRNA and microRNA signatures may ultimately provide the most robust strategy for diagnosis and risk stratification in HFpEF and other fibrotic cardiac conditions.

## 7 Therapeutic Potential of circRNAs in Cardiac Fibrosis

### 7.1 Therapeutic Targeting Strategies

#### 7.1.1 Inhibition Approaches

Several approaches have been developed for targeting circRNAs therapeutically. RNA interference represents a primary strategy, utilizing either short interfering RNA (siRNA) or short hairpin RNA (shRNA) targeting the unique back‐splice junction of circRNAs [114]. The CRISPR/Cas9 system offers another approach by disrupting the pairing of introns flanking the circularized exons, though this presents more challenges than RNAi‐based strategies [114]. The newer CRISPR/Cas13 system, particularly RfxCas13d, has shown superior knockdown efficiency with minimal effects on linear counterparts [114]. Experimental evidence supports the effectiveness of these approaches, with studies demonstrating successful circRNA silencing using siRNAs [110, 115]. For instance, siRNA‐mediated knockdown of specific circRNAs has shown effectiveness in suppressing cell growth and reducing colony formation [115]. CircHIPK3, derived from Exon 2 of the HIPK3 gene, has increased expression in heart tissues and CFs after angiotensin II treatment. Silencing it decreases cardiac fibrosis area, improves diastolic function, inhibits cardiac fibroblast proliferation and migration, decreases the function of TGF‐β, downregulates alpha‐SMA, COL1A1, and COL3A1 expression by sponging miR‐29b‐3p [97, 98, 99, 100].

#### 7.1.2 Replacement and Gene Augmentation Strategies

In addition to inhibitory approaches, gene therapy approaches aimed at restoring or augmenting beneficial circRNA activity are emerging as a promising strategy for modulating cardiac fibrosis. These include the potential to induce or suppress circRNA expression in specific tissues through vector‐based delivery or transfection of purified, in vitro–generated circRNAs [116]. Local injection of cholesterol‐conjugated RIG‐I si‐circRNA has shown promising results in experimental hepatocellular carcinoma models, particularly in enhancing treatment sensitivity [117]. Compared to conventional gene therapy approaches, circRNA‐based therapeutics offer several theoretical advantages, including enhanced molecular stability and the potential to modulate multiple regulatory pathways. Among these approaches, synthetic circRNAs mimetics represent a compelling platform for restoring the function of anti‐fibrotic circRNAs that are pathologically downregulated during cardiac remodeling.

##### 7.1.2.1 Synthetic circRNA Mimetics: Gain‐of‐Function and Replacement Strategies

Another emerging and arguably more transformative therapeutic paradigm involves the exogenous delivery of synthetic circRNA mimetics, engineered circular RNA molecules designed to replace or augment the function of endogenous circRNAs that are pathologically downregulated in cardiac fibrosis. Several circRNAs with established anti‐fibrotic properties, such as circNFIB, circYAP, circMACF1, and Circ_LAS1L, are significantly downregulated during cardiac remodeling [87, 88, 89, 91]. The loss of these protective circRNAs removes critical molecular brakes on fibroblast activation, ECM deposition, and pro‐fibrotic signaling. Synthetic mimetics that restore the expression or function of these molecules, therefore, represent a conceptually distinct and complementary therapeutic strategy [118]. Synthetic circRNA production has advanced considerably through the development of efficient in vitro circularization methods, including group I intron‐based autocatalytic splicing, T4 RNA ligase‐mediated ligation, and chemical synthesis with ribozyme‐assisted circularization [119, 120]. These techniques now allow the production of highly pure, sequence‐defined circRNA molecules with controlled secondary structures. Compared to linear mRNA, synthetic circRNAs offer inherent advantages, including markedly extended half‐life due to resistance to exonuclease‐mediated degradation, absence of free 5′ and 3′ ends that would otherwise trigger innate immune sensors, and the capacity for sustained protein expression when engineered to contain IRES or m6A modifications [65].

One major application is the design of synthetic circRNAs that recapitulate the miRNA sponging activity of downregulated endogenous circRNAs. For example, synthetic analogs of circNFIB, which normally sequester miR‐433 to limit fibroblast proliferation, could restore the inhibitory tone on this pro‐fibrotic miRNA axis. Similarly, synthetic versions of circMACF1, which suppresses CF activation via the miR‐16‐5p/SMAD7 axis, could attenuate TGF‐β‐driven fibrosis at the post‐transcriptional level [87, 88]. Engineering multiple MREs into a single synthetic circRNA scaffold could further broaden anti‐fibrotic coverage by simultaneously sponging multiple pro‐fibrotic miRNAs [121]. Perhaps the most revolutionary application of synthetic circRNA technology lies in their engineering as translational platforms for producing therapeutic peptides or proteins in situ. As discussed in Section Results, 3, some endogenous circRNAs naturally encode functional peptides via IRES‐driven or m6A‐facilitated cap‐independent translation [15, 68, 69, 70]. Building on this biology, synthetic circRNAs can be engineered to harbor IRES elements upstream of therapeutic open reading frames, enabling efficient production of anti‐fibrotic peptides directly within cardiac cells following delivery. For instance, synthetic circRNAs encoding dominant‐negative fragments of TGF‐β receptor components, SMAD pathway inhibitors, or anti‐fibrotic peptides such as those derived from the Myo9a‐208 sequence could directly suppress fibroblast activation and collagen synthesis in a highly localized manner [122]. Importantly, because the same circular RNA molecule simultaneously acts as a regulatory sponge and a translational template, synthetic circRNA platforms offer a unique dual‐function therapeutic modality unavailable with conventional linear RNA or small‐molecule approaches [123]. The therapeutic potential of this approach has been demonstrated in recent proof‐of‐concept studies. Wesselhoeft et al. showed that synthetic circRNAs with optimized IRES elements produce protein for significantly longer durations than equivalent linear mRNAs in both cell culture and in vivo models, with reduced immunogenicity compared to unmodified linear RNA [124]. Furthermore, the capacity to introduce chemical modifications such as pseudouridine (Ψ), 5‐methylcytidine (m5C), or N1‐methylpseudouridine (m1Ψ) into synthetic circRNA backbones without disrupting circularization has enabled further reduction of innate immune activation while preserving translational efficiency [121]. These advances collectively position synthetic circRNA mimetics as a next‐generation platform for cardiac gene therapy, complementary to the inhibition strategies already discussed.

#### 7.1.3 Pathway‐Directed Therapeutic Interventions

Recent pharmacological insights have positioned the circRNA–GREM1 axis as a key target in fibroproliferative diseases. As a potent antagonist of Bone Morphogenetic Protein (BMP) signaling, GREM1 facilitates a permissive environment for TGF‐β–mediated myofibroblast activation and ECM deposition [125]. Emerging evidence suggests that this GREM1‐driven pathological process is amenable to circRNA‐mediated intervention. Specifically, pro‐fibrotic circRNAs may act as upstream regulators of the GREM1/BMP rheostat by sequestering microRNAs that would otherwise suppress GREM1 expression. Through this mechanism, circRNAs indirectly attenuate the protective, anti‐fibrotic effects of BMP signaling, thereby promoting myocardial stiffness and fibrotic progression. In line with this mechanism, therapeutic strategies such as anti‐GREM1 antibodies and circRNA‐based gene modulation have been proposed as means of selectively targeting this pathway. Compared with conventional TGF‐β inhibitors, which are often limited by systemic toxicity, modulation of circRNA–GREM1 interactions may offer a more selective approach to controlling pathological remodeling while preserving reparative processes [125]. These findings highlight the potential of circRNA‐based therapeutic interventions to provide targeted and context‐specific strategies for the treatment of cardiac fibrosis, while underscoring the need for further investigation into their safety, specificity, and clinical applicability.

### 7.2 Delivery Systems: The Major Translational Bottleneck in circRNA Therapeutics

The therapeutic efficacy of circRNA‐based interventions, whether inhibitory or replacement‐based, is fundamentally constrained by the challenges of efficient, targeted, and safe in vivo delivery. CircRNAs are large macromolecules that cannot passively diffuse across cell membranes, and naked circRNAs administered systemically are susceptible to immune recognition and rapid clearance. Two principal delivery platforms have emerged as leading candidates for circRNA therapeutics in cardiac fibrosis: lipid nanoparticles (LNPs) and extracellular vesicles (EVs). Each platform offers distinct mechanistic advantages and faces unique translational limitations that must be carefully considered in the development of cardiac‐targeted therapies [126].

#### 7.2.1 LNPs

LNPs are currently the most clinically advanced RNA delivery platform, having been validated at scale through the global deployment of mRNA‐LNP COVID‐19 vaccines. Their application to circRNA delivery represents a natural and well‐supported extension of this technology. The circRNA‐LNP platform has demonstrated higher stability and more durable protein expression compared to linear mRNA‐LNP formulations in preclinical models, attributable to the inherent resistance of circular RNA to exonuclease degradation following endosomal escape [124]. Structurally, LNPs are composed of an ionizable lipid that facilitates endosomal escape via protonation at acidic pH, a phospholipid helper lipid, cholesterol, and a polyethylene glycol (PEG)‐lipid that stabilizes the particle and prolongs circulation time. LNPs offer several critical advantages for circRNA delivery. First, their formulation can be optimized to encapsulate large RNA cargo with high efficiency, protecting the circRNA from nuclease degradation and immune recognition in circulation. Second, ionizable LNP formulations facilitate efficient endosomal escape, releasing functional circRNA into the cytoplasm. Third, LNP composition can be chemically tuned using selective organ‐targeting (SORT) technology, in which the inclusion of specific lipid components directs biodistribution toward the liver, lung, or spleen, with recent formulations achieving measurable cardiac tropism [127]. Fourth, LNPs are amenable to scalable good manufacturing practice (GMP) production, a prerequisite for clinical translation [126]. Despite these advantages, significant challenges remain. LNPs exhibit inherent hepatotropism due to apolipoprotein E (ApoE)‐mediated uptake, limiting their efficiency for cardiac delivery following systemic administration. While intramyocardial or pericardial injection could circumvent this limitation, such routes are invasive and clinically impractical for chronic fibrotic disease. Immunogenicity remains a concern: while synthetic circRNAs are intrinsically less immunogenic than linear RNA, the LNP carrier itself can activate innate immune pathways via Toll‐like receptor signaling, potentially exacerbating the inflammatory milieu already present in fibrotic myocardium [120]. Furthermore, repeated LNP administration can induce anti‐PEG antibodies, reducing delivery efficiency over time, a critical limitation for chronic conditions like cardiac fibrosis that may require sustained therapeutic intervention [128].

#### 7.2.2 EVs

EVs, comprising exosomes (30–150 nm) and microvesicles (100–1000 nm), represent a biologically derived delivery platform with distinct advantages over synthetic nanoparticles, particularly for cardiac applications. EVs are naturally produced by virtually all cell types and play physiological roles in intercellular communication, including in cardiac remodeling and fibrosis [129]. Their endogenous origin confers low immunogenicity, inherent biocompatibility, and the ability to cross biological barriers that resist synthetic nanoparticles. Crucially, EVs can be engineered to carry circRNA cargo, exploiting their natural membrane‐enclosed architecture to protect RNA payloads from degradation [130]. EVs offer several compelling advantages for circRNA delivery to the fibrotic heart. Their phospholipid bilayer membrane closely mimics the cell membrane, facilitating efficient fusion and cargo release. Unlike LNPs, EVs do not require ionizable lipids or chemical modifications to achieve endosomal escape, relying instead on natural membrane‐mediated fusion pathways. EVs derived from cardiomyocytes or cardiac progenitor cells have been shown to preferentially home to injured myocardial tissue through surface expression of integrins and adhesion molecules such as ICAM‐1 and tetraspanins, offering an inherent degree of cardiac tropism [129]. Engineering EV surfaces with cardiac‐targeting ligands, including anti‐myosin antibodies, cardiac homing peptides, or folate receptor ligands, further enhances myocardial specificity. Furthermore, EVs can carry both RNA and protein cargo simultaneously, enabling co‐delivery of anti‐fibrotic circRNAs alongside cardioprotective proteins [130]. The translational development of EV‐based circRNA delivery faces significant manufacturing and regulatory challenges. EVs are produced in relatively small quantities by donor cells, and current bioreactor‐based production methods remain insufficient for clinical‐scale manufacturing. Heterogeneity in EV size, surface composition, and cargo loading between batches represents a major obstacle to reproducibility and regulatory approval. Loading exogenous circRNA into EVs with high efficiency is technically demanding; current approaches include electroporation, sonication, and co‐incubation, each with trade‐offs between loading efficiency and EV integrity. Additionally, EVs may display donor‐cell surface antigens that could trigger alloimmune responses when derived from allogeneic sources, necessitating autologous production or extensive surface engineering for broad clinical application [118].

#### 7.2.3 Emerging and Alternative Delivery Approaches

Beyond LNPs and EVs, several alternative delivery modalities are under active investigation. Adeno‐associated virus (AAV)‐based delivery of circRNA expression cassettes offers the prospect of long‐term, tissue‐specific circRNA expression following a single administration, exploiting the well‐characterized cardiac tropism of AAV serotypes 6, 8, and 9. However, the large size of circRNA expression cassettes, combined with AAV's limited packaging capacity (~4.7 kb), imposes constraints on the complexity of circRNAs that can be delivered via this route. Cationic polymer‐based nanoparticles, including polyethylenimine (PEI) and poly(lactic‐co‐glycolic acid) (PLGA) formulations, have demonstrated efficacy in delivering small RNA molecules to cardiac tissue and may be adaptable for larger circRNA payloads with further optimization. Local delivery strategies, including intramyocardial injection of cholesterol‐conjugated circRNA and pericardial instillation of nanoparticle‐formulated circRNA, circumvent systemic barriers and reduce off‐target distribution, and have shown promising results in preclinical MI and pressure‐overload models [117]. These approaches may be particularly relevant for post‐surgical cardiac fibrosis prevention, where direct access to the myocardium is available [128].

## 8 Future Perspectives

### 8.1 Research Directions

Despite significant progress in understanding circRNA biology, several key research priorities must be addressed to advance the field toward clinical translation.

#### 8.1.1 Emerging Technologies

Recent technological advancements have revolutionized the study of circRNAs in cardiac fibrosis. High‐throughput sequencing technologies, such as RNA‐seq, have been instrumental in identifying circRNA expression profiles and elucidating their roles in cardiac fibrosis across various models [82]. Additionally, single‐cell RNA sequencing has emerged as a powerful tool to uncover circRNA heterogeneity at the cellular level, which is crucial for understanding their context‐dependent regulatory functions [79].

The advent of CRISPR‐based technologies has facilitated precise manipulation of circRNAs, such as selectively targeting back‐splice junctions (BSJs) without affecting the parent linear RNAs [131]. Furthermore, circRNA‐specific probes and advanced imaging techniques could enhance our understanding of circRNA‐protein interactions, which play pivotal roles in the progression of cardiac fibrosis [132]. Such technological innovations will accelerate discoveries in circRNA biology and foster new therapeutic strategies. Future studies should apply these technologies to systematically map circRNA expression across different stages of cardiac fibrosis and heart failure phenotypes, particularly using patient‐derived tissues and longitudinal clinical samples.

In parallel, advances in synthetic biology are accelerating the development of therapeutic circRNA manufacturing. Improvements in in vitro transcription fidelity, ribozyme‐assisted circularization efficiency, and purification methodologies are progressively reducing the cost and increasing the yield of GMP‐grade synthetic circRNAs, addressing one of the key barriers to clinical translation. Ribosome profiling (RIBO‐seq) and proteomics approaches are further enabling the systematic identification of endogenous circRNAs with translational potential, expanding the catalog of circRNA‐encoded peptides relevant to fibrosis biology [65].

#### 8.1.2 Computational Predictions

Computational biology and bioinformatics approaches are essential for predicting circRNA interactions and regulatory networks. Tools such as miRanda and TargetScan have been widely used to map circRNA‐miRNA‐mRNA interaction networks, enabling the identification of key axes in fibrotic pathways [82]. Machine learning algorithms are also increasingly employed to predict circRNA‐associated protein interactions and their potential functional outcomes [133]. Moreover, advancements in ribosome profiling and codon‐optimized databases could aid in identifying translatable circRNAs, unveiling new aspects of their coding potential [134]. These computational approaches complement experimental studies, allowing researchers to explore the therapeutic relevance of circRNAs in cardiac fibrosis [135]. Future research should integrate computational prediction tools with experimental validation to identify circRNA‐centered regulatory networks driving fibroblast activation and ECM remodeling.

#### 8.1.3 Interdisciplinary Research Opportunities

The study of circRNAs in cardiac fibrosis demands a multidisciplinary approach, integrating molecular biology, clinical research, and computational sciences [109]. For instance, proteomics and metabolomics could be used alongside transcriptomics to uncover the broader impact of circRNA‐mediated regulation [109]. Collaborative efforts between cardiovascular researchers and computational biologists could improve the identification of disease‐specific circRNA signatures, facilitating their clinical translation [82]. Additionally, the exploration of circRNAs in other fibrotic diseases, such as liver or kidney fibrosis, could reveal conserved mechanisms and potential therapeutic overlaps [109].

In addition to their regulatory functions, emerging evidence suggests that certain circRNAs can encode functional peptides, revealing an underexplored dimension of circRNA biology in cardiac fibrosis [65, 136, 137]. For example, circ_0036176, derived from the Myo9a gene, encodes the peptide Myo9a‐208 and has been shown to suppress cardiac fibroblast proliferation via modulation of the cyclin/Rb pathway in heart failure models [122]. Similarly, circNlgn encodes the nuclear‐localized Nlgn173 protein, which alters the transcription of fibrotic genes like ING4 and SGK3, exacerbating cardiac remodeling and fibrosis [77]. In doxorubicin‐induced cardiotoxicity models, circNlgn also activated DNA damage pathways and inflammatory cascades through interaction with H2AX, leading to adverse structural remodeling [76].

These findings underscore a critical translational dimension of circRNAs, where their encoded peptides may functionally contribute to disease processes. However, the field remains in its infancy due to challenges in detecting and validating circRNA‐derived proteins. Expanding interdisciplinary research into circRNA translation—especially using emerging tools like RIBO‐seq and artificial circRNA synthesis—could offer transformative insights and therapeutic possibilities for cardiac fibrosis and beyond [134, 135, 138, 139]. Interdisciplinary research will be essential to translate circRNA discoveries into clinically actionable insights.

### 8.2 Clinical Translation

Before circRNA‐based therapies can be implemented clinically, several critical translational challenges must be addressed.

#### 8.2.1 Translational Research Considerations

Despite significant preclinical advancements, the clinical translation of circRNA‐based therapeutics remains a challenge. One major hurdle is achieving specificity in targeting circRNAs without off‐target effects, especially given their overlap with linear RNAs [114]. Emerging strategies such as BSJ‐specific RNA interference and CRISPR/Cas13‐based systems could help address this limitation. Beyond molecular targeting, important pharmacological and safety considerations remain. Although the covalently closed structure of circRNAs confers enhanced stability [140], additional investigation is required to better characterize their long‐term distribution, immune compatibility and repeated‐dose safety profiles in vivo. Preclinical models, such as genetically engineered circRNA mice, may be essential for testing the safety and efficacy of circRNA‐based interventions [129]. Regulatory considerations specific to circRNA therapeutics are also evolving. As circular RNA molecules do not naturally integrate into the genome and are degraded without permanent genomic modification, they may attract a more favorable regulatory profile compared to DNA‐based gene therapies. However, standardized quality control metrics for synthetic circRNA, including circularization efficiency, RNA integrity, residual linear RNA content, and immunostimulatory potential, are still being developed and will require harmonization across regulatory agencies before clinical trials can proceed [141].

#### 8.2.2 Potential Clinical Applications

Future therapeutic strategies may exploit circRNAs both as diagnostic biomarkers and as therapeutic targets for modulating fibrotic signaling pathways. For example, circRNA‐Uck2 has been shown to distinguish acute MI from unstable angina, highlighting its diagnostic utility [142]. Similarly, circRNAs such as circSMAD4 and circARAP1 have been implicated in the regulation of fibrotic pathways, making them attractive therapeutic targets [74, 84]. Therapeutic applications of circRNAs extend beyond RNA interference. CircRNA‐derived peptides, produced via cap‐independent translation, offer new opportunities for modulating fibrotic signaling cascades [76, 77]. These novel approaches could complement existing pharmacological therapies for cardiac fibrosis.

Combination strategies that pair circRNA‐based therapeutics with existing anti‐fibrotic pharmacology, including angiotensin‐converting enzyme inhibitors, mineralocorticoid receptor antagonists, and SGLT2 inhibitors, warrant systematic investigation. circRNA delivery could, in principle, sensitize fibrotic myocardium to these agents by restoring anti‐fibrotic regulatory networks that pharmacological agents cannot directly engage. Furthermore, circRNA‐derived peptides produced via engineered synthetic circRNA platforms introduce an entirely new category of cardiac biologics, combining the delivery advantages of RNA therapeutics with the functional diversity of protein‐based drugs [65].

#### 8.2.3 Personalized Medicine Implications

CircRNAs' tissue‐specific, disease‐specific, and temporal expression profiles make them ideal candidates for personalized medicine [143, 144]. By leveraging circRNA sequencing data, clinicians could develop individualized treatment regimens targeting specific fibrotic pathways [140]. Personalized approaches might include delivering patient‐specific circRNA inhibitors or mimics using EVs or nanoparticle carriers [94]. Moreover, understanding circRNA polymorphisms could aid in identifying patient populations more likely to benefit from circRNA‐targeted therapies [82]. The combination of advanced sequencing technologies and machine learning could enable the development of predictive models for patient stratification, further enhancing the precision of circRNA‐based interventions [82].

## 9 Conclusion

As a major contributor to heart failure, cardiac fibrosis represents a major global health burden with limited diagnostic biomarkers and treatment methods. With their unique structure as covalently closed‐loop ncRNA, circRNA‐based strategies have emerged as promising therapeutic approaches through influencing ECM deposition, inflammatory processes, genetic modulation, and fibroblast activation. Additionally, because of their stability in circulation, they serve as important and reliable biomarkers, opening the door for more accurate diagnostic measures. Preclinical studies suggest that modulating circRNAs through increasing or decreasing their expression via siRNA, CRISPR/Cas13, EVs, or LNPs can decrease remodeling. Despite their promising outlook, challenges in cell‐targeted delivery and evasion of immunogenicity remain. Additionally, the understanding of their functional mechanisms remains unclear. Overall, the advancements in circRNAs open a new door to therapeutic modalities in cardiology, though further research integrating molecular biology, clinical trials, and computational sciences is needed to support their broad clinical application. Computational approaches could help outline circRNA's role in fibrotic pathways, enhancing the literature's understanding of its pathogenicity, ultimately enhancing its therapeutic potential and improving patient outcomes.

## Author Contributions

Conceptualization of Ideas: Toufik Abdul‐Rahman, Mrinmoy Kundu. Data curation: Toufik Abdul‐Rahman. Visualization: Toufik Abdul‐Rahman. Supervision: Toufik Abdul‐Rahman, Rahul Gupta, Carl J. Lavie. Writing of initial draft: Poulami Roy, Jann Ludwig Mueller‐Gomez, Aditya Gaur, Mohammad Alabdulrahman, Hala Ibrahim Thaalibi, Mrinmoy Kundu, Bibek Shrestha, Syeda Asna Shireen Munawer, Emmanuella Ifeoma Okonkwo, Ayaan Hassan Mohamed, Riddhi Mondal, Rakhshanda Khan, Enaholo Tobechukwu Mathew Olumese. Writing ‐ Review & editing: Toufik Abdul‐Rahman, Rahul Gupta, Carl J. Lavie.

## Funding

The authors have nothing to report.

## Ethics Statement

The authors have nothing to report.

## Consent

All authors approve the current version of the manuscript for publication.

## Conflicts of Interest

The authors declare no conflicts of interest.

## Data Availability

The authors have nothing to report.
